# Supplementary material for: Plasma FGF21 Levels Are Not Associated with Weight Loss or Improvements in Metabolic Health Markers upon 12 Weeks of Energy Restriction: Secondary Analysis of an RCT
Source: Nutrients. 2022 Nov 28;14(23):5061. doi: 10.3390/nu14235061 (PMC9735516; doi:10.3390/nu14235061)
Supplement: Supplementary file 1 [file nutrients-14-05061-s001.zip › nutrients-1943218-supplementary.pdf]

Supplementary materials

Table S1. Correlations between both fasting plasma FGF21 and the postprandial response of plasma FGF21 and markers of metabolic health

	**Fasting plasma FGF21**			**Postprandial plasma FGF21 response[b]**		
	Spearman's ρ	p	n	Spearman's ρ	p	n
Body weight	-0.07	0.469	98	-0.09	0.376	97
BMI	0.03	0.786	98	-0.07	0.491	97
Waist circumference	-0.03	0.789	98	-0.09	0.397	97
Visceral AT	-0.06	0.638	69	**-0.24**	**0.046**	69
Subcutaneous AT	0.09	0.447	69	0.09	0.443	69
VAT/SAT ratio[a]	-0.08	0.491	69	-0.21	0.081	69
IHL[a]	-0.04	0.734	79	-0.03	0.781	79
HbA1c	0.02	0.810	98	-0.03	0.773	97
Fasting glucose	0.09	0.367	98	**-0.24**	**0.020**	97
iAUC glucose[a]	-0.01	0.953	94	-0.00	0.966	93
Fasting insulin[a]	-0.01	0.905	98	**-0.33**	**0.001**	97
iAUC insulin[a]	0.08	0.457	98	-0.13	0.204	97
Fasting triglycerides[a]	0.02	0.830	98	-0.001	0.992	97
Fasting total cholesterol	0.10	0.315	98	0.07	0.487	97
Fasting HDL cholesterol	0.07	0.486	98	-0.03	0.766	97
Fasting FFAs[a]	**-0.22**	**0.031**	96	-0.14	0.182	95
HOMA-IR[a]	0.00	0.985	98	**-0.34**	**0.001**	97
ALAT	0.14	0.172	98	-0.14	0.169	97
ASAT[a]	0.15	0.151	98	**-0.23**	**0.021**	97
γGT[a]	-0.06	0.578	98	0.03	0.756	97

[a] Log2 transformed to improve normality

[b] Calculated as the change from plasma FGF21 in the fasted state to 120 minutes after the high-fat mixed meal

Abbreviations: BMI, body mass index; AT, adipose tissue; IHL, intra-hepatic lipid content; HbA1c, glycated hemoglobin A1c; iAUC, incremental area under the curve; HDL, high-density lipoprotein; FFAs, free fatty acids; HOMA-IR, homeostatic model assessment of insulin resistance; ALAT, aminotransferase; ASAT, aspartate aminotransferase; γGT, gamma-glutamyl transferase.

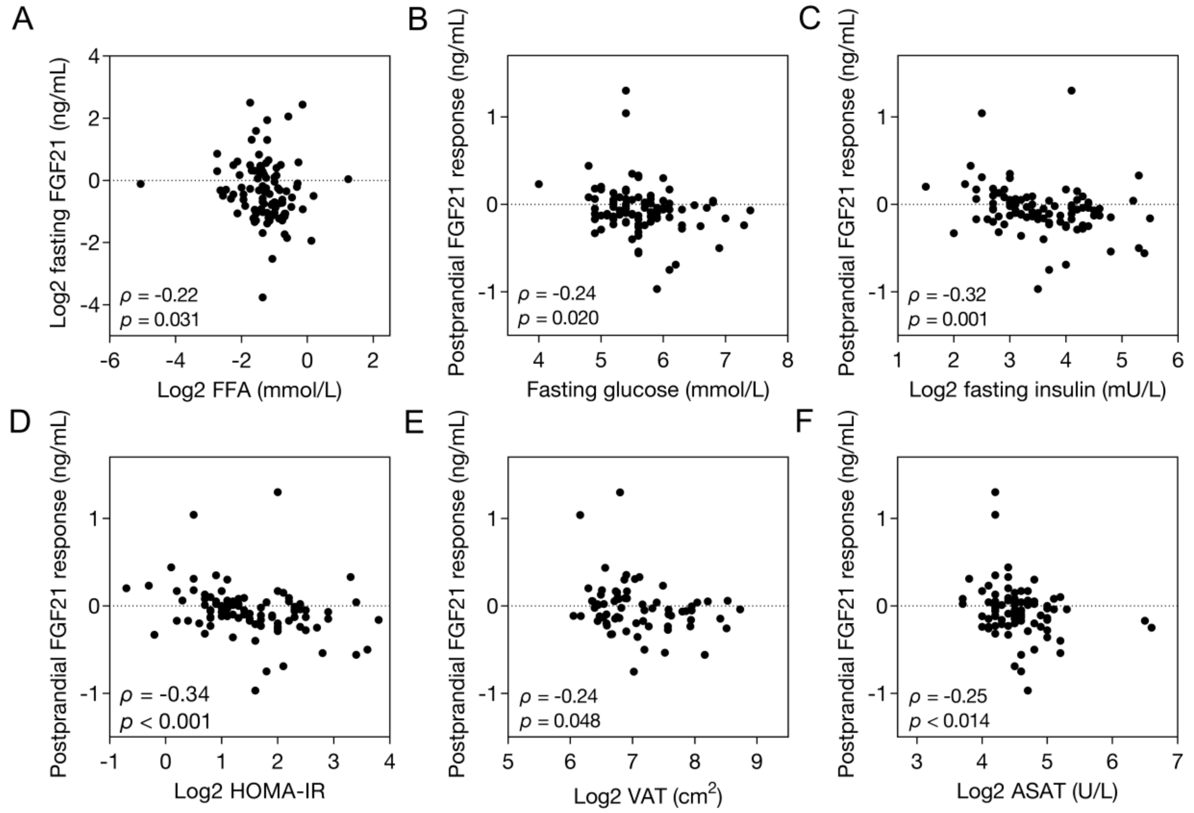

Figure S1. Scatter plots of fasting plasma FGF21 and the postprandial plasma FGF21 response upon a liquid high-fat mixed meal with markers of metabolic health. Plasma free fatty acids (FFA) were inversely correlated to fasting plasma FGF21 (**A**). Fasting plasma glucose (**B**), fasting plasma insulin (**C**), homeostatic model assessment of insulin resistance (HOMA-IR) (**D**), visceral adipose tissue (VAT) (**E**), and serum aspartate aminotransferase (ASAT) (**F**) were inversely correlated to the postprandial plasma FGF21 response. The postprandial FGF21 response was calculated as the change from plasma FGF21 in the fasted state to 120 minutes after the high-fat mixed meal. ρ denotes the Spearman correlation coefficient.

Table S2. Correlations between the change in fasting plasma FGF21 and the change in metabolic health markers upon a 12-week diet

	**Δ Fasting plasma FGF21**								
	**Control group**			**Low-nutrient-quality diet**			**High-nutrient-quality diet**		
Δ	Spearman's *ρ*	*p*	*n*	Spearman's *ρ*	*p*	*n*	Spearman's *ρ*	*p*	*n*
Body weight	-0.13	0.535	26	-0.01	0.931	38	0.05	0.799	34
BMI	-0.13	0.540	26	0.04	0.833	38	0.01	0.968	34
Waist circumference	-0.10	0.642	26	-0.07	0.670	37	0.01	0.964	33
VAT	-0.06	0.837	16	0.20	0.311	27	0.36	0.074	26
SAT	0.14	0.602	16	0.09	0.650	27	**-0.49**	**0.012**	26
VAT/SAT ratio	-0.06	0.820	16	0.15	0.458	27	0.36	0.072	26
IHL	-0.44	0.058	19	-0.04	0.829	33	0.15	0.428	29
HbA1c	**-0.43**	**0.029**	26	0.24	0.143	38	0.06	0.741	34
Fasting glucose	**0.40**	**0.044**	26	0.24	0.147	38	0.15	0.403	34
iAUC glucose	-0.08	0.696	26	0.07	0.694	38	0.32	0.063	34
Fasting insulin	0.02	0.919	26	0.17	0.320	38	0.21	0.250	33
iAUC insulin	-0.10	0.643	26	-0.10	0.568	38	0.29	0.096	34
Fasting triglycerides	-0.04	0.860	26	0.09	0.586	38	-0.11	0.530	34
Fasting total cholesterol	-0.07	0.751	26	0.06	0.704	38	-0.06	0.748	34
Fasting HDL cholesterol	-0.01	0.948	26	0.10	0.560	38	-0.14	0.438	34
Fasting FFAs	0.04	0.850	26	-0.09	0.580	38	-0.26	0.157	31
HOMA-IR	0.09	0.675	26	0.20	0.231	38	0.22	0.227	33
ALAT	0.28	0.170	26	-0.14	0.416	38	0.16	0.371	34
ASAT	0.24	0.229	26	-0.18	0.271	38	0.13	0.461	34
γGT	-0.24	0.232	26	-0.27	0.101	38	0.13	0.460	34

Abbreviations: BMI, body mass index; AT, adipose tissue; IHL, intra-hepatic lipid content; HbA1c, glycated hemoglobin A1c; iAUC, incremental area under the curve; HDL, high-density lipoprotein; FFAs, free fatty acids; HOMA-IR, homeostatic model assessment of insulin resistance; ALAT, aminotransferase; ASAT, aspartate aminotransferase; γGT, gamma-glutamyl transferase.

Table S3. Correlations between change in the postprandial plasma FGF21 response upon a liquid high-fat mixed meal and the change in metabolic health markers upon a 12-week diet

	**Δ Postprandial plasma FGF21 response[a]**								
	**Control group**			**Low-nutrient-quality diet**			**High-nutrient-quality diet**		
Δ	Spearman's *ρ*	*p*	*n*	Spearman's *ρ*	*p*	*n*	Spearman's *ρ*	*p*	*n*
Body weight	0.30	0.131	26	-0.17	0.316	37	-0.02	0.905	32
BMI	-0.29	0.151	26	0.14	0.399	37	-0.03	0.879	32
Waist circumference	-0.33	0.098	26	-0.05	0.752	36	-0.07	0.697	31
VAT	-0.19	0.478	16	0.31	0.124	26	0.22	0.294	25
SAT	-0.12	0.656	16	-0.06	0.774	26	-0.45	0.023	25
VAT/SAT ratio	0.09	0.753	16	0.28	0.165	26	0.24	0.250	25
IHL	0.06	0.814	19	0.18	0.323	32	0.09	0.665	28
HbA1c	**-0.42**	**0.031**	26	0.01	0.958	37	-0.26	0.154	32
Fasting glucose	0.24	0.239	26	0.18	0.288	37	0.16	0.388	32
iAUC glucose	-0.28	0.162	26	0.13	0.460	37	0.29	0.111	32
Fasting insulin	-0.35	0.077	26	**0.35**	**0.034**	37	0.14	0.454	31
iAUC insulin	-0.21	0.296	26	0.16	0.347	37	0.23	0.201	32
Fasting triglycerides	0.25	0.226	26	0.18	0.280	37	**-0.36**	**0.041**	32
Fasting total cholesterol	0.23	0.260	26	0.09	0.576	37	-0.12	0.504	32
Fasting HDL cholesterol	0.19	0.340	26	-0.25	0.131	37	0.10	0.596	32
Fasting FFAs	0.11	0.583	26	-0.17	0.304	37	-0.20	0.309	29
HOMA-IR	-0.28	0.166	26	0.32	0.054	37	0.17	0.351	31
ALAT	0.39	0.050	26	0.07	0.699	37	0.00	0.998	32
ASAT	0.35	0.084	26	-0.12	0.476	37	0.03	0.871	32
γGT	-0.08	0.694	26	0.10	0.555	37	-0.09	0.622	32

[a] Calculated as baseline vs. post-intervention difference in the change from plasma FGF21 in the fasted state to 120 minutes after the high-fat mixed meal
Abbreviations: BMI, body mass index; AT, adipose tissue; IHL, intra-hepatic lipid content; HbA1c, glycated hemoglobin A1c; iAUC, incremental area under the curve; HDL, high-density lipoprotein; FFAs, free fatty acids; HOMA-IR, homeostatic model assessment of insulin resistance; ALAT, aminotransferase; ASAT, aspartate aminotransferase; γGT, gamma-glutamyl transferase

Table S4. Ranges and means of tertiles of habitual nutrient intake, alcohol consumption, and sweet-taste preference

	**Tertile 1**			**Tertile 2**			**Tertile 3**		
	Range	Mean	SD	Range	Mean	SD	Range	Mean	SD
Fat, en%	26.4 – 36.8	33.5	2.7	36.9 – 40.4	38.6	1.2	40.8 – 54.5	44.8	3.8
Protein, en%	11.7 – 15.0	13.8	0.8	15.0 – 16.6	15.8	0.5	16.7 – 27.9	18.7	2.3
Carbohydrates, en%	23.5 – 37.2	32.9	3.4	37.5 – 41.3	39.5	1.1	41.4 – 50.3	45.2	2.9
Mono- and disaccharides, en%	4.6 – 16.1	13.4	2.5	16.3 – 19.5	18.0	0.9	19.6 – 30.0	23.1	2.7
Polysaccharides, en%	11.7 – 19.8	16.7	2.1	20.0 – 22.6	21.3	0.8	22.7 – 31.0	25.2	2.0
Alcohol, en%	0.0 – 1.1	0.3	0.4	1.1 – 4.7	2.7	1.1	4.9 – 14.6	6.8	2.3
Sweet-taste preference, AU	2.0 – 2.6	2.4	0.2	2.6 – 2.8	2.7	0.1	2.8 – 3.3	3.0	0.1

Abbreviations: SD, standard deviation; en%, % of daily energy intake
